# FAPI-PET/CT in Cancer Imaging: A Potential Novel Molecule of the Century

**DOI:** 10.3389/fonc.2022.854658

**Published:** 2022-05-25

**Authors:** Rong Huang, Yu Pu, Shun Huang, Conghui Yang, Fake Yang, Yongzhu Pu, Jindan Li, Long Chen, Yunchao Huang

**Affiliations:** ^1^ Department of PET/CT Center, Yunnan Cancer Hospital, The Third Affiliated Hospital of Kunming Medical University, Cancer Center of Yunnan Province, Kunming, China; ^2^ Medical Imaging Key Laboratory of Sichuan Province, North Sichuan Medical College, Nanchong, China; ^3^ Department of Nuclear medicine, Nanfang Hospital, Southern Medical University, Guangzhou, China; ^4^ Department of Thoracic Surgery I, Yunnan Cancer Hospital, The Third Affiliated Hospital of Kunming Medical University, Cancer Center of Yunnan Province, Kunming, China

**Keywords:** fibroblast-activating protein, FAPI, PET, Neoplasms, imaging, 18 F-FDG

## Abstract

Fibroblast activation protein (FAP), a type II transmembrane serine protease, is highly expressed in more than 90% of epithelial tumors and is closely associated with various tumor invasion, metastasis, and prognosis. Using FAP as a target, various FAP inhibitors (FAPIs) have been developed, most of which have nanomolar levels of FAP affinity and high selectivity and are used for positron emission tomography (PET) imaging of different tumors. We have conducted a systematic review of the available data; summarized the biological principles of FAPIs for PET imaging, the synthesis model, and metabolic characteristics of the radiotracer; and compared the respective values of FAPIs and the current mainstream tracer ^18^F-Fludeoxyglucose (^18^F-FDG) in the clinical management of tumor and non-tumor lesions. Available research evidence indicates that FAPIs are a molecular imaging tool complementary to ^18^F-FDG and are expected to be the new molecule of the century with better imaging effects than ^18^F-FDG in a variety of cancers, including gastrointestinal tumors, liver tumors, breast tumors, and nasopharyngeal carcinoma.

## Introduction

Malignant tumors are the second leading cause of patient death worldwide (1). The tumor stroma is an important component of the tumor microenvironment (TME), where cancer cell-autonomous mutations (and other alterations) combine with changes in the tumor stroma to drive tumorigenesis, progression, metastasis, and treatment resistance formation. The tumor stroma consists of specialized connective tissue cells, including fibroblasts, mesenchymal stromal cells (MSCs), osteoblasts, and chondrocytes, as well as the extracellular matrix (ECM). Cells in the non-malignant stroma are usually quiescent and maintain a balance between the ECM and the epithelial cell zone. When cancer develops, when cancer develops, the stroma undergoes vast changes to become fibrotic and activated, and fibroblasts and MSCs become more proliferative, secreting higher levels of growth factors, cytokines, and chemokines, with fibroblasts acting as sentinel cells that often begin to activate and accumulate early in the lesion; fibroblasts are called cancer-associated fibroblasts (CAFs), myofibroblasts are further differentiated fibroblasts that are present during wound healing or other processes that cause matrix remodeling, and in cancer, and CAFs often resemble myofibroblasts due to the presence of activated stroma (2). Fibroblast activation protein (FAP), which is highly expressed in CAFs but largely absent in normal tissues, is one of the specific markers of CAFs in TME and is closely related to tumor invasion, metastasis, angiogenesis, and prognosis and is considered an essential target for the diagnosis and treatment of malignant tumors (3–5). We searched PubMed, MEDLINE, and Scopus databases for one or more combinations of the following terms: “cancer-associated fibroblasts”, “fibroblast activating protein”, “FAPI”, “FAPI-PET”, “fibroblast activation protein inhibitor”, “FAPI”, and “FAPI-PET”. All English-language papers in the search return results were evaluated and included if they fell within the scope of this review.

## FAP Biological Properties

Originally known as the F19 antigen, FAP is a type II transmembrane serine protease consisting of 761 amino acids in a dimer consisting of an α subunit of 95 kDa and a β subunit of 105 kDa with collagenase activity that breaks down gelatin and type I collagen as well as a dipeptidyl peptidase (DPP)–like activity (6). FAP belongs to the DPP subfamily, which includes DPP4, quiescent cell proline dipeptidase (QPP), FAP, prolyl oligopeptidase (POP), DPP8, DPP, DPP6, and DPP10 (7). Among them, FAP has 50% amino acid sequence homology and 70% homology of catalytic structural domain with DPP4, and they can form heterodimerization complexes to synergistically regulate the growth, differentiation, adhesion, and metastasis of tumor cells (8). FAP is highly expressed in the stroma surrounding more than 90% of epithelial-derived tumors and their metastases and is consistently expressed in bone and soft tissue tumor cells. It may also be transiently expressed in fetal mesenchymal tissues, healing wounds after endometrial detachment in adults, and organ reconstruction sites; however, it is usually not expressed in normal tissues, benign lesions, and precancerous tissues (9, 10). Studies have shown that a large amount of tumor mesenchyme (50%–90%) is present in most malignant lesions, and high expression of FAP is closely associated with invasion, metastasis, and poor prognosis of these malignant tumors, suggesting that FAP is promising as an essential target for malignant tumor imaging and treatment (11, 12).

## Targeting CAFs for Imaging

Imaging studies of malignancies targeting FAP are performed by linking FAP inhibitors (FAPIs) or FAP antibodies to radionuclides using chelators followed by positron emission tomography/computed tomography (PET/CT) imaging, PET/magnetic resonance imaging, or single-photon emission computed tomography (SPECT) imaging. Much of the early work on FAPIs focused on pyrrolidine-2-boronic acid derivatives, of which the first pyrrolidine-2-boronic acid derivative to be clinically tested was Val-boroPro (Talabostat, PT-100), which exhibited a significant affinity for one or more members of the DPP subfamily members but are not very selective for FAP (13). In addition, a small-molecule antibody B12 IgG targeting the extracellular structural domain of FAP is being used in preclinical studies for prostate imaging, showing that the considerable molecular weight of the FAP antibody results in slow clearance *in vivo*, resulting in high background signal and limited sensitivity for focal detection (14). Small-molecule FAPIs (UAMC-1110) based on the 4-quinolinyl-glycyl-2-cyanopyrrolidine framework overcome the disadvantages of pyrrolidine-2-boronic acid derivatives FAP antibody B12 IgG due to their simultaneous nanomolar affinity for FAP and high selectivity (15). Much of the current work in FAPI research has focused on the chemical modification of quinoline-based small-molecule inhibitors to obtain derivatives with higher affinity for FAP and superior pharmacokinetics. Several derivatives have shown promising clinical results, including FAPI-02 (16), FAPI-04 (17), FAPI-46 (18), FAPI-34 (19), FAPI-74 (20), DOTA.SA.FAPI and DATA5m.SA.FAPI (21), DOTA-2P(FAPI)_2_ (22), and FAPI-42 (23). Many derivatives are being developed, and some promising preclinical data have been obtained, such as ^18^F-fluoroglycosylation-FAPI (^18^F-FGlc-FAPI) (24), QCP01 and QCP02 (25), and AAZTA5.SA.FAPI (26) (**Table 1**).

**Table 1:** FAPIs that have been used in clinical studies.

Tracer	Chemical Structure	Author and Year	Tumor (Patients)	Key Finding
**FAPI-02**	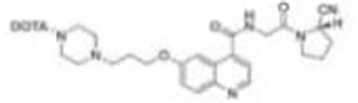	Loktev et al. (16) 2018	Metastasized breast cancer (one patient) metastasized lung cancer (one patient) metastasized pancreatic cancer (one patient)	^68^Ga-FAPI-02 is seen to selectively accumulate in FAP-expressing tissue and to be significantly higher than^18^F-FDG in malignant lesions(SUVmax of 13.3); unlike ^18^F-FDG, ^68^Ga-FAPI-02 shows no uptake in brain, spleen, or liver.
**FAPI-04**	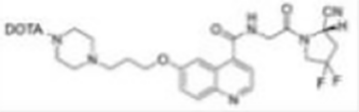	Lindner et al. (17) 2018	Metastasized breast cancer (two patients)	FAPI-04 shows rapid internalization into FAP-positive tumors and fast clearance from the body, resulting in very fast accumulation at tumor sites (10 min after tracer administration); the effective tumor uptake after 24 h—100% higher for FAPI-04 than for FAPI-02.
**FAPI-46**	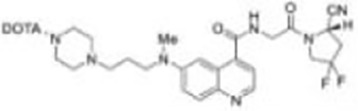	Loktev et al. (18) 2019	Various cancers (eight patients)	High intratumoral uptake of FAPI-46 already 10 min after administration; demonstration of higher tumor uptake with FAPI-46 Than with FAPI-04.
**FAPI-34**	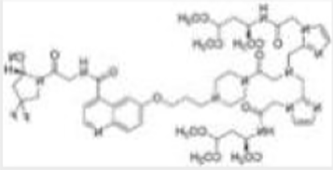	Lindner et al. (19) 2020	Metastasized ovarian cancer (one patient) and pancreatic cancer (one patient)	^99m^Tc-FAPI-34 represents a powerful tracer for diagnostic scintigraphy, especially when PET imaging is not available.
**FAPI-74**	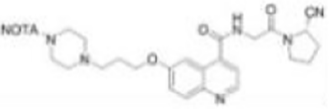	Giesel et al. (20) 2021	Lung cancer (10 patients)	The high contrast and low radiation burden compared with ^18^F-FDG; best correlation to CT-based target volumes occurred at an SUV of three-fold the background.
**DOTA-2P(FAPI)2**	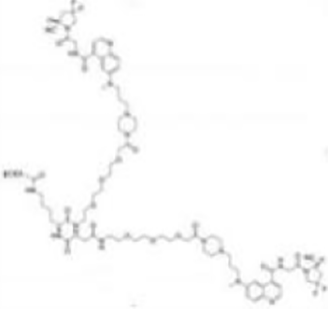	Zhao et al. (22) 2021	Nasopharyngeal cancer, papillary thyroid cancer, and HCC (three patients)	Exhibits better *in vivo* pharmacokinetics and higher tumor uptake than FAPI alone; has a long retention time in the blood pool and shows higher physiological uptake in the thyroid and pancreas.
**DOTA.SA.FAPI**	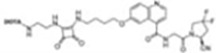	Ballal et al. (27) 2021	Various cancers (54 patients)	TBR was comparable to previously reported data for FAPI-04; there was an advantage in brain metastasis compared with ^18^F-FDG.
**NOTA-FAPI-04** **(FAPI-42)**	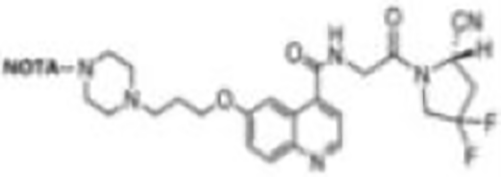	Wang et al. (23) 2021	Various cancers (10 patients)	No defluorination during the assay time; higher uptake and TBR in liver tumors and bone tumors compared with ^68^Ga-FAPI-04.

### FAPIs Automated Production Technology

Jiang et al. attempted to label NOTA-FAPI-04 with [^18^F]AlF, the radioactivity per batch was 9.095 ± 0.587 GBq and the radiochemical yield (RCY) was 26.4 ± 1.5%, and the radiotracer obtained by the automated synthesis equipment was used for PET/CT imaging of a patient with liver metastases from invasive cholangiocarcinoma (IDC). The images showed high tumor-to-background uptake ratios (TBRs) of 8.44 (28). Dahl et al. successfully produced good manufacturing practice (GMP)–compliant ^18^F-FAPI-74 in the GE TRACERlab FX2N module and the cassette-based module Trasis AllInOne (AIO) in radioactivity per batch of 6410 MBq and 6900 MBq, respectively, with the RCY of about 20% (29). Naka et al. performed a proof-of-concept automated synthesis of [^18^F] AlF-FAPI-74 with one-pot and one-step by a multi-purpose synthesizer CFN-MPS200; the radioactivity per batch was 11.3 ± 1.1 GBq, and the RCY was 37.0% ± 4.3% (30).

### ^68^Ga-Labeled FAPIs

FAPIs coupled to radionuclide 68Ga *via* DOTA chelator are mainly FAPI-04, FAPI-46, FAPI-74, DOTA-2P(FAPI)_2_, DOTA.SA.FAPI, and DATA5m.SA.FAPI.

**Figure 1:**
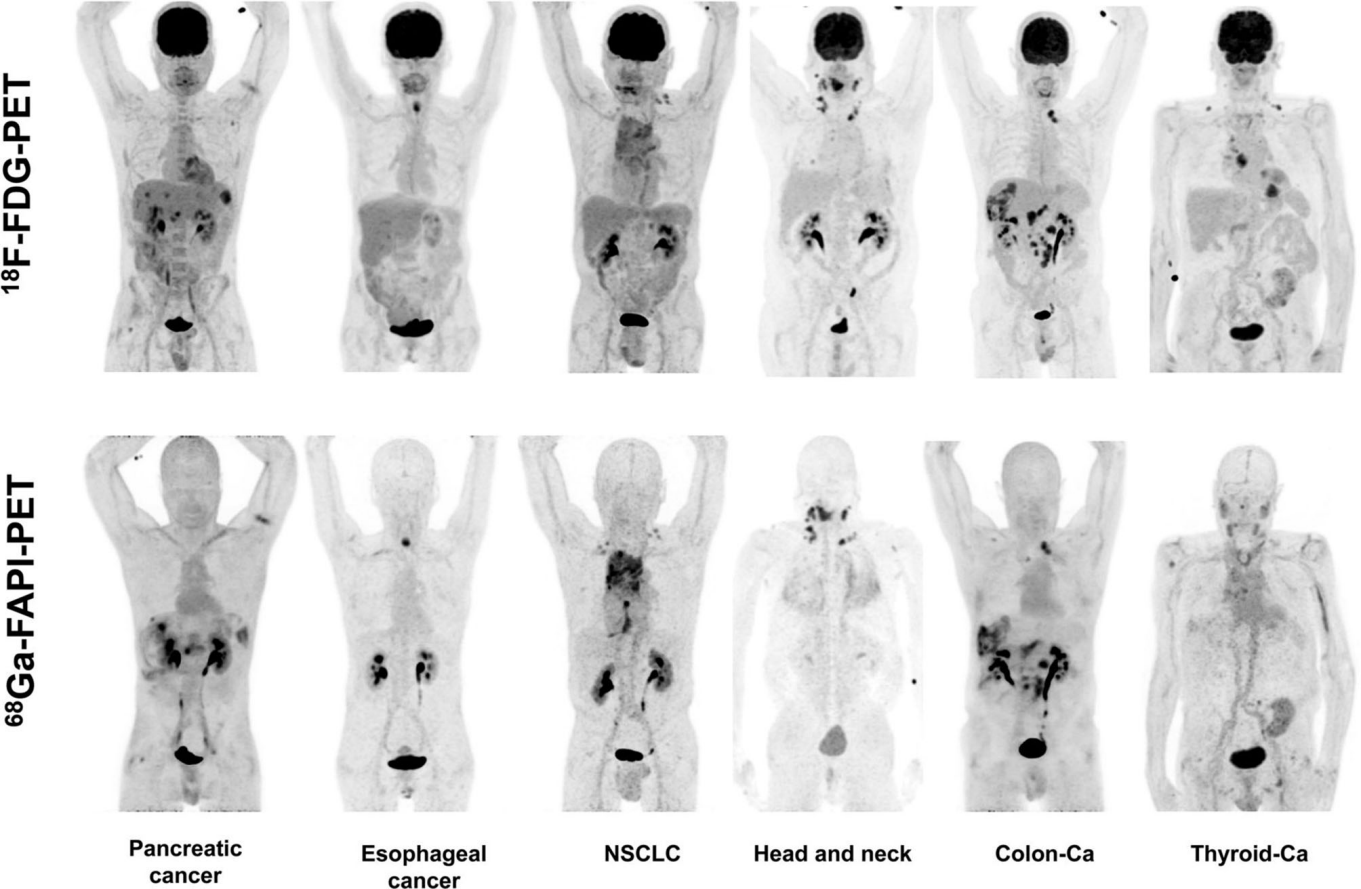
Six patients underwent intra-individual comparison of ^18^F-FDG-PET and ^68^Ga-FAPI-PET imaging over a 9-day period. Five of the six patients showed similar high tumor uptake in ^18^F-FDG-PET and ^68^Ga-FAPI-PET, and three of the six patients had more lesions detected due to low background uptake in the visceral or pharyngeal mucosa. Patients with iodine uptake negative thyroid cancer exhibited low ^68^Ga-FAPI tracer uptake compared with ^18^F-FDG-PET; Ca, cancer; NSCLC, non–small cell lung cancer (31).

FAPI-04 is a small-molecule FAPI derivative that has been chemically modified to provide better imaging results, with effective doses of 1.64E-02mSv/MBq. Kratochwil et al. performed ^68^Ga-FAPI-02 and ^68^Ga-FAPI-04 PET/CT imaging on 80 patients and obtained uptake data for tracers of 28 types of tumors (31). Among them, sarcoma, esophageal, breast, cholangiocarcinoma, and lung cancer showed high uptake rates with Maximum Standardized Uptake Value (SUVmax) >12; liver tumors and colorectal tumors, head and neck tumors, pancreatic tumors, ovarian tumors, and prostate tumors had moderate uptake rates (6 < SUVmax < 12). Pheochromocytoma, renal cell carcinoma, differentiated thyroid cancer, adenoid cyst, and gastric cancer had the lowest uptake rates (SUVmax < 6). The images from this study were named “Image of the Year” at the 2019 North American Nuclear Medicine Annual Meeting (**Figure 1**). Giesel et al. analyzed the biodistribution and preliminary dosimetric data of ^68^Ga-FAPI-02 and ^68^Ga-FAPI-04 in 50 patients containing 14 different types of tumors (32). Compared with ^18^F-Fludeoxyglucose (^18^F-FDG), ^68^Ga-FAPI showed lower uptake in the brain, liver, and oral/pharyngeal mucosa and no significant difference in tumor and other normal tissues, showing higher TBRs. Wang et al. performed the first biodistribution and internal radiation dosimetry after ^68^Ga-FAPI-04 injection in six Chinese adults, and the results were in general agreement with the findings of Giesel et al. (32, 33).

FAPI-46 is another FAPI derivative with highly desirable imaging results, with effective doses of 7.80E-03mSv/MBq. The initial clinical imaging study work with FAPI-46 included eight patients, and compared with FAPI-04, ^68^Ga-FAPI-46 had a higher TBR and has a longer tumor retention time, making it ideal for tumor imaging and targeted therapy (18). After that, a study by Meyer et al. showed that ^68^Ga-FAPI-46 PET/CT has good dosimetric properties with high TBR increasing over time (34).

Zhao Let al. synthesized DOTA-2P(FAPI)_2_ with two FAPI-46 monomers (22). ^68^Ga-DOTA-2P(FAPI)_2_ has an effective dose of 1.19E-02mSv/MBq, higher than that of ^68^Ga-FAPI-46 and comparable to ^68^Ga-FAPI-02 (1.80E-02mSv/MBq) and ^68^Ga-FAPI-04. Intra-tumoral uptake of ^68^Ga-DOTA-2P(FAPI)_2_ is higher than that of ^68^Ga-FAPI-46 (SUVmax of 8.1–39.0 vs. 1.7–24.0) in most tumor lesions in nasopharyngeal carcinoma, papillary thyroid carcinoma, and hepatocellular liver cancer. The above studies suggest that FAPI dimers exhibit better *in vivo* pharmacokinetics and higher tumor uptake than FAPI monomers, leading to a more apparent contrast of primary lesions and metastases with normal tissue, thus potentially improving the sensitivity of lesion detection. However, the tracer showed higher physiological uptake in the thyroid and pancreas and prolonged retention time in the blood pool. The data from this study need to be validated with a larger sample size.

Moon et al. reported FAPI derivatives DOTA.SA.FAPI and DATA5m.SA.FAPI with IC_50_ of 0.7–1.4 nM; SA replaces the heterocyclic nitrogen portion of FAPI-04 to form a squaramide unit. The chelators used were macrocyclic DOTA and the hybrid chelator DATA5m (21). In animal studies, ^68^GaDOTA.SA.FAPI had good TBR, comparable to the previously reported animal data for FAPI-04 (21). In clinical studies, ^68^Ga-DATA5m.SA.FAPI PET/CT showed specific uptake in focal nodular hyperplasia in the liver (35), and there was no significant difference in tumor uptake between 68Ga-DOTA.SA.FAPI and ^18^F-FDG compared with ^18^F-FDG in 14 types of tumor imaging in a total of 54 patients, with the exception of brain metastatic lesions (27). Next, the team found that radiolabeling SA.FAPI with AAZTA5 as a chelator (i.e., AAZTA5.SA.FAPI) was superior to both DOTA and DATA5m in terms of labeling process and drug yield, and the IC_50_ of the product was in the low nanomolar range (0.55–0.57 nM), but the drug is still in preclinical studies (26).

### ^18^F-labeled FAPIs

With the development of ^18^F production equipment and labeling technology, the advantages of ^18^F clinical application will gradually increase: first, the maximum β^+^ energy of ^18^F is significantly lower than that of ^68^Ga, so the sensitivity and spatial resolution of ^18^F-PET imaging is better than ^68^Ga-PET imaging, which can improve the image contrast to a certain extent; second, ^68^Ga has become expensive due to a sharp increase in global demand and thus a shortage of supply (36); in addition, the ^68^Ge/^68^Ga generator is not yet capable for mass production of ^68^Ga due to technical limitations, which means that the automated production of large quantities of FAPIs radiotracers is limited. In an automated synthesis study of ^68^Ga-FAPI-46 by Spreckelmeyer et al., it was found that the radioactivity at the end of the synthesis was only 700–1,700 MBq, which was only sufficient for the examination of four to six patients (37). In comparison, the long half-life of ^18^F (1.13 h) supports centralized commercial production and distribution to customers with no ^18^F production facilities, resulting in a significant reduction in production costs (38). The development of low-power benchtop cyclotrons has made ^18^F dispersion production easier (automated, low cost, and on-demand production); the ^18^F labeling of peptides has become more efficient due to the development of chelator and labeling technologies. Currently, in FAPI radiotracer research, an increasing number of researchers are experimenting with ^18^F labeling of FAPIs to develop radiopharmaceuticals that can meet the global demand for large-scale assays, especially in developing countries. For example, ^18^F-FAPI-74 and ^18^F-NOTA-FAPI-04 showed good visualization in clinical studies, and ^18^F-FGlc-FAPI-04 showed superior performance in *in vitro* experiments (20, 23, 24).

**Figure 2:**
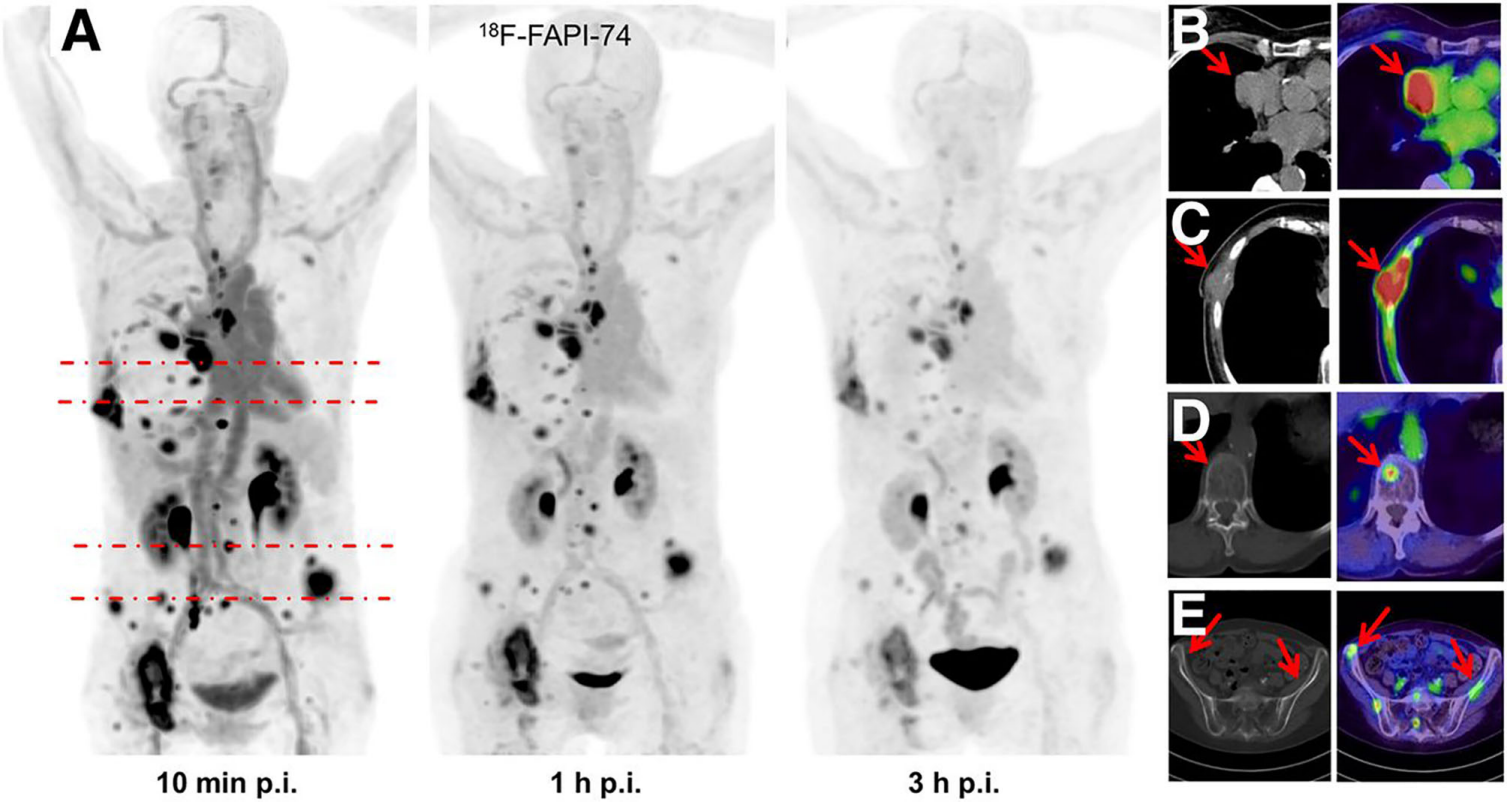
One patient with non–small cell lung cancer who developed systemic metastasis. **(A)** Maximum intensity projections at 10 min, 1 h, and 3 h after injection of ^18^F-FAPI-74 PET. **(B)** The uptake of ^18^F-FAPI-74 was significantly different between tumor and normal myocardium. **(C)** FAPI-positive lesions were confirmed on CT examination. **(D, E)** Some bone metastases were detected only by FAPI PET. All highlighted arrows represent FAPI uptake in relation to morphology (20).

Giesel et al. applied ^18^F-FAPI-74 and ^68^Ga-FAPI-74 for the first time in clinical practice and successfully attached [^18^F]AlF to FAPI *via* 1,4,7-triazacyclonane-*N*,*N*′,*N*′-triacetin acid (NOTA) for the first time and showed satisfactory imaging results. A total of 10 patients with non–small cell lung cancer in this study received ^68^Ga-FAPI-74 and ^18^F-FAPI-74 imaging within 1 week. The data showed that the best tumor-to-background contrast presents 1 h after injection, and the estimated radiation dose of ^18^F-FAPI-74 was lower than that of other ^18^F-labeled PET scanner tracers, and there was no difference in tumor uptake between lung adenocarcinoma and lung squamous cell carcinoma. This study shows the value of ^18^F-FAPI-74 for lung cancer diagnosis and detection of small lung cancer lesions, but the number of cases is too small to analyze whether it is useful for staging and radiotherapy planning guidance (20) (**Figure 2**). Others have attempted to label multiple FAPIs with [^18^F] AlF and found that ^18^F-FAPI-74 gave the most favorable results in an *in vitro* study, and they performed a visualization study in a patient with metastatic non–small cell lung cancer and obtained conclusions similar to those of Giesel et al. (20, 39).

^18^F-NOTA-FAPI-04 (^18^F-FAPI-42) was obtained by replacing the DOTA with chelator NOTA in FAPI-04 and Aqueous^18^F-Labeling by the Al^18^F chelation technique (21, 40). ^18^F-FAPI-42 was initially used to diagnose lung, pancreatic, colorectal, prostate, and lymphoma and was compared with ^18^F-FDG (21). The results showed that the excretion route of ^18^F-FAPI-42 was mainly in the urinary and biliary systems, similar to the previously reported tracers ^18^F-FAPI-74, ^68^Ga-FAPI-04, and ^68^Ga-FAPI-46; compared with ^18^F-FDG imaging, ^18^F-FAPI-42 had lower SUVmean in the liver, brain, spleen, and bone marrow; in addition, ^18^F-FAPI-42 bone uptake was low, indicating that this tracer was not defluorinated at the time of detection. Another study including a total of 22 patients with hepatocellular carcinoma, lung cancer, colorectal cancer, gastric cancer, esophageal cancer, cheek carcinosarcoma, abdominal neuroendocrine carcinoma, and clear cell carcinoma of the bladder conducted a human biodistribution study of ^18^F-FAPI-42 and compared it with ^68^Ga-FAPI-04 (40). Data from this study showed that the optimal image acquisition time for ^18^F-FAPI-42 was 1 h after injection; ^68^Ga-FAPI-42 showed higher physiological uptake in the parotid, salivary gland, thyroid, and pancreas compared with ^68^Ga-FAPI-04; both had equal detection rates for lesions; in addition, ^18^F-FAPI-42 showed higher TBRs in the liver, bone, lymph nodes, pleura, and peritoneal metastases.

^18^F-FGlc-FAPI-04 is an [^18^F]-6-fluoro-6-deoxyglycosylated FAPI radiotracer with an IC_50_ of 167 nM, much higher than ^68^Ga-FAPI-04 (32 nM), showing higher tumor uptake and longer tracer clearance times were shown in HT1080-xenografts and U87MG-xenografts. However, because the tracer is mainly eliminated *via* the hepatobiliary pathway and sees intraosseous and intra-articular uptake, this has hindered the entry of the tracer into clinical studies (24).

### ^99m^Tc and ^111^In-Labeled FAPIs

FAPI-34, QCP01[1-(6-((3-((4-((2-((S)-2-cyanopyrrolidin-1-yl)-2-oxoethyl)-carbamoyl)quinolin-6-yl)oxy)propyl)amino)-6-oxohexyl)-2-((E)-2-((E)-3-(2-((E)-3,3-dimethyl-5-(trioxidaneylthio)-1-(4-(trioxidaneylthio)butyl)indolin-2-ylidene)ethylidene)-2-(4-(trioxidaneylthio)phenoxy)cyclohex-1-en-1-yl)vinyl)-3,3-dimethyl-3H-indol-1-ium-5-sulfonate)] and QCP02(2,2′,2″-(10-(1-carboxy-4-((3-((4-((2-((S)-2-cyanopyrrolidin-1-yl)-2-oxoethyl)carbamoyl)quinolin-6-yl)oxy)propyl)amino)-4-oxobutyl)-1,4,7,10-tetraazacyclododecane-1,4,7-triyl)triacetic acid) both demonstrated low nanomolar inhibition (19, 25). FAPI-34, with an IC_50_ of 6.4–12.7 nM, has been used for SPECT imaging of patients with ovarian and pancreatic cancer metastases, showing good imaging results. When PET imaging is not possible due to equipment, ^99m^Tc-FAPI-34 can be used as the imaging agent for SPECT imaging, which can also be used for diagnosis and efficacy evaluation (19). QCP01 and QCP02 were applied in tumor transplantation models with higher tumor and normal organ uptake than FAPI-02 and FAPI-04, indicating that QCP01 and QCP02 have high affinity for FAP. In addition, ^111^In-QCP02 SPECT imaging showed higher tumor uptake and lower kidney uptake, which is still in the preclinical study stage (25).

### Comparison of FAPI-Specific PET Imaging With FDG in Malignant Neoplasm

**Figure 3:**
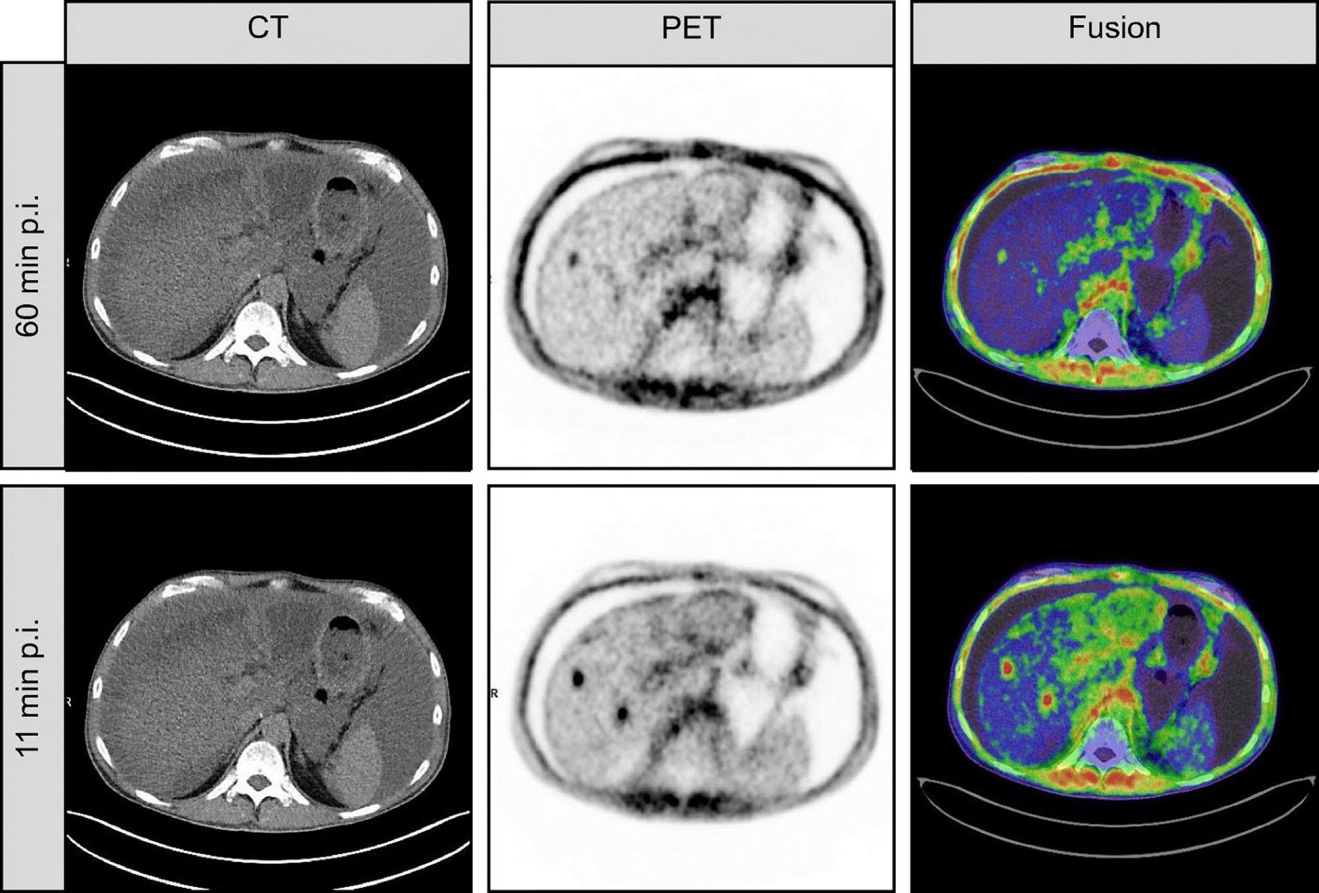
This is a case of inconsistent early and late imaging in a 54-year-old patient with metastatic pancreatic cancer. Re-staging was performed with FAPI-46 PET after resection of the primary focus. FAPI-46 PET performed 11 min after injection showed two intrahepatic metastases, one of which was not detected at 60 min of imaging (41).

With FAPI-specific PET imaging, patients do not require dietary preparation, and high-quality images can be obtained early (10 min to 1 h) after tracer injection (20, 41) (**Figure 3**).

**Figure 4:**
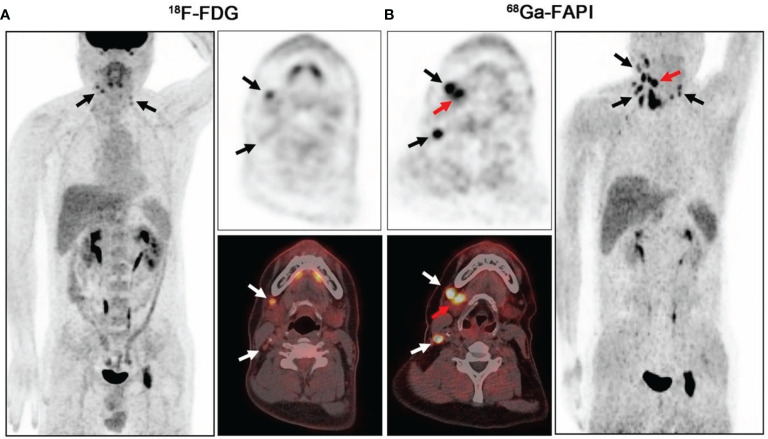
PET/CT scans of ^18^F-FDG **(A)** and ^68^Ga-FAPI **(B)** in a 41-year-old patient with lymph node metastasis from salivary gland ductal carcinoma. ^18^F-FDG-PET/CT was negative for the primary tumor. On ^68^Ga-FAPI-PET/CT, the right submandibular gland shows strong uptake (**B**, red arrow; SUVmax = 15.80), whereas the left submandibular gland has low background uptake (SUVmax = 6.87) (black and white arrows indicate lymph node metastases) (44).

In head and neck tumors, compared with ^18^F-FDG, it was able to improve the staging, target area contour, and prognostic assessment of oral squamous carcinoma, nasopharyngeal carcinoma, and adenoid cystic carcinoma. For example, in 45 patients with nasopharyngeal carcinoma, the detection rates of primary and metastatic foci by FDG were 97.0% and 75.2%, respectively, and the detection rates of primary and metastatic foci by FAPI-04 were 100% and 95.0%, respectively (42); FAPI-PET/CT detected more details of primary foci and lymph node metastases, resulting in a change in staging in 5 of 12 cases of adenoid cystic carcinoma (43). Among 18 patients with head and neck tumors in whom primary lesions were not detected by FDG PET/CT imaging, FAPI PET/CT detected primary lesions in 5 of them (44). However, FAPI may not be advantageous in detecting lymph nodes and distant metastases (41, 44) (**Figure 4**).

In malignant brain lesions, cranial MRI is currently the primary imaging method for diagnosis, staging, and outlining radiotherapy target areas for high-grade gliomas in the brain. However, PET/CT and PET/MRI often provide more tumor progression and prognostic assessment (45). Conventional ^18^F-FDG tracers have high physiological uptake in the brain, resulting in low TBRs and obscuring accurate information about the tumor. FAPI has low physiological uptake in the brain and is highly expressed on the cell surface of high-grade gliomas (excluding diffuse astrocytomas). Clinical studies have shown high uptake of FAPI imaging agents in high-grade gliomas (SUVmax = 2.8 ± 0.6), whereas low-grade gliomas showed only mild uptake (SUVmax = 0.35 ± 0.10) (46, 47). Therefore, FAPI imaging agents may be helpful in the detection and target area sketching of high-grade gliomas in the brain, non-invasive grading of gliomas, and assessment of prognosis after radiotherapy. In addition, it showed higher TBR for brain metastases from gastric, breast, lung, and liver cancers, with a higher detection rate than^18^F-FDG (47–50).

FAPI PET/CT shows high uptake and high TBRs in primary and lymph node metastases of non–small cell lung cancer, and the detection rate of lymph node metastases is higher than ^18^F-FDG and thus may improve the staging of lung cancer and contribute to the outline of radiotherapy target areas (20, 50).

^18^F-FDG PET and PET/CT are less sensitive for detecting regional lymph node metastases in esophageal squamous cell carcinoma (about 66%) (51). Because of the high expression of FAP in esophageal cancer, FAPI PET shows a high uptake rate (SUVmax > 12) and high TBR in esophageal cancer and its metastases, and lymph node metastases of esophageal cancer with a false-negative ^18^F-FDG test are strongly positive by FAPI test, which improves the detection rate of lymph node lesions (31). The high TBR and high sensitivity of FAPI for primary foci and lymph node metastases provide more information for clinical purposes, both to improve the staging of esophageal cancer and to help outline the target volume (GTV) in precision radiotherapy. ^18^F-FDG has limitations in detecting certain pathological types of gastric malignancies, such as non-mesenchymal diffuse carcinoma, mucinous carcinoma, and indolent cell carcinoma (52, 53). In contrast, in some small sample studies, although FAPI showed mostly low uptake in gastric cancer, it also showed high TBR, which may still bring a turnaround for PET/CT detection of the abovementioned tumors (31). For example, in a study that included 10 patients with primary gastric tumors, the detection rates of FDG and FAPI were 50% (5/10) and 100% (10/10), respectively (47). In addition, it has been suggested that FAPI appears to be the best performing imaging agent for PET imaging of liver malignancies at present. Because of the high non-specific uptake of ^18^F-FDG in the liver, the false-negative rate of ^18^F-FDG PET in detecting HCCs is high, about 40%–50% (54). In a study of 25 patients with suspected primary liver malignancies (hepatocellular carcinoma/intrahepatic cholangiocarcinoma) on ^68^Ga-FAPI-04 imaging, the detection rate of primary liver malignancies was higher with FAPI than with ^18^F-FDG (96% vs. 65%) (55). Another study that included 20 patients with suspected primary malignancy of the liver also showed a higher sensitivity of FAPI for the diagnosis of malignancy (100% vs. 58.8%) (56).

**Figure 5:**
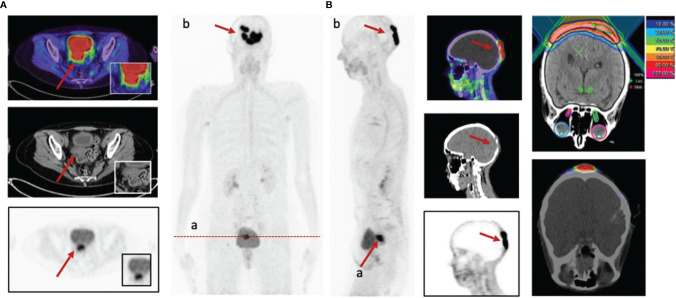
A 58-year-old patient with cervical cancer underwent ^68^Ga-FAPI-PET/CT staging prior to radiotherapy. recurrence of cervical cancer (SUVmax = 14.4) **(A)**. In addition, intracranial metastases showed strong FAPI uptake (SUVmax = 32.3) **(B)**, which allowed for precise delineation of radiotherapy target areas (57).

Breast carcinoma (15%), ovarian carcinoma (5%), and uterine carcinoma (4%) are the most common gynecologic malignancies, and accurate staging is essential for treatment guidance (1). The most commonly used radiotracer for PET/CT diagnosis of gynecologic malignancies is ^18^F-FDG. A multicenter prospective study including 31 gynecologic malignancies showed higher TBRs in breast, ovarian, cervical, endometrial, and smooth muscle metastases; uterine smooth muscle sarcoma; and fallopian tube cancer. FAPI PET had higher TBRs in metastatic lesions than ^18^F-FDG [regional lymph node metastases (31.9 vs. 27.4) and distant metastases (13.0 vs. 5.7)]. However, there were significant differences in FAPI uptake between premenopausal and postmenopausal patients in the endometrium (mean SUVmax of 11.7 vs. 3.0) and breast (mean SUVmax of 1.8 vs. 1.0), which may affect the detection of lesions at these sites (57) (**Figure 5**).

In a case report of liver metastasis from malignant melanoma, the lesion showed moderate ^68^Ga-FAPI-04 uptake (SUVmax of 6.5) but was not compared with the current PET standard tracer ^18^F-FDG (58). In another retrospective analysis of FAPI PET/CT imaging of rare malignancies, patients with melanoma (one case) also showed more favorable imaging results (59).

In conclusion, FAPI-specific tracers simplify the clinical workflow and have significant advantages in detecting malignancies in the brain, liver, and other sites.

### Diagnostic Efficacy of FAPI and FDG in Non-Neoplastic Lesions

IgG_4_-related disease (IgG_4_-RD) is an autoimmune inflammatory disease that occurs mainly in the pancreas, biliary tract, salivary glands, kidneys, aorta, and other organs, and the disease is often accompanied by infiltration of IgG_4_+ cells or (and) activated fibroblasts (60). A study that included 26 patients with IgG_4_-RD showed that ^68^Ga-FAPI PET/CT detected an additional ^18^ (13.2%) involved organs out of 136 involved organs in 26 patients compared with ^18^F-FDG PET/CT and had a higher positive rate than ^18^F-FDG PET/CT in detecting pancreatic, bile duct/liver, and lacrimal gland involvement, but the former was at a disadvantage in detecting lymph node involvement (60). Co-imaging with ^68^Ga-FAPI-04 and ^18^F-FDG-PET may enable non-invasive tracking of the evolution of IgG_4_-RD from inflammation to fibrosis (60, 61). In a FAPI PET/CT imaging of patients with early-stage immune checkpoint inhibitor (ICI)–associated myocarditis, the heart showed specific uptake (62). High tracer accumulation was seen in the left ventricular myocardium on FAPI PET/CT imaging in a patient undergoing chemotherapy for pancreatic cancer. These study data may provide new ideas for early diagnosis of myocardial injury and intervention (63). In a case of thyroiditis with ^68^Ga-FAPI PET/CT, the uptake rate of inflammatory lesions (SUVmax of 4.2) was similar to that of hypofractionated thyroid cancer (SUVmax < 6), suggesting that FAPI PET/CT has difficulty in differentiating hypofractionated thyroid cancer from benign lesions (31, 64). Monitoring the activity of aortitis often requires a joint assessment of imaging data, clinical data, and biological data, which are not always correlated, making the work challenging. A patient with aortitis underwent ^68^Ga-FAPI-PET/CT, showing mild thickening of the walls of the bilateral carotid, subclavian, and thoracoabdominal aorta, where there is no specific uptake of ^18^F-FDG. Therefore, ^68^Ga-FAPI-PET/CT may be superior to ^18^F-FDG in monitoring the activity of aortitis (65). In two case reports, the TB lesions showed high uptake of the FAPI tracer with higher intensity than ^18^F-FDG; this helps in assessing extra-pulmonary tuberculosis lesions (66, 67).

### FAPI Tracer Imaging Findings Correlate With Histopathology

The intensity of FAPI imaging agent uptake by various tumors is closely related to the degree of FAP expression, e.g., breast cancer and esophageal cancer, where FAP expression levels are high and tumor uptake of FAPI imaging agent is very high (SUVmax >12) (9, 31). The non-specific uptake of FAPI visualizers also correlates with the FAP expression profile, with FAP expression levels often upregulated during stromal remodeling, as in wound healing, and these sites tend to exhibit higher FAPI visualizer uptake (68, 69). Interestingly, cancers such as colon and pancreatic cancers, which have the highest desmoplastic reaction by histopathology, exhibit only moderate 68Ga-FAPI uptake (SUVmax of 6–12), this may be due to the variability of cancer, i.e., cancer development is complex and FAP expression varies at different stages of stromal remodeling, a conjecture that requires large sample studies to verify (31, 70).

### Limitations and Perspectives of FAPIs

Stromal remodeling occurs not only in malignancies but also in development, wound healing, chronic inflammation (e.g., arthritis, atherosclerotic plaques, and fibrosis), and certain physiologic processes where FAP is often highly expressed, posing challenges for differentiating benign from malignant, tumor and peritumor chronic inflammation in FAPI imaging (71). For example, a clinical study including 92 patients found high nonspecific uptake of FAPI-04 and FAPI-46 at sites where scarring (SUVmax of 7.7 ± 3.3) or degenerative lesions (SUVmax of 7.7 ± 2.9) occurred in patients, i.e., these sites may show false-positive results on tumor detection (69); one patient with pancreatic cancer who developed chronic peritumor inflammation due to radiotherapy was unable to identify the target volume for radiotherapy with FAPI tracer (72). In addition, some sites with low or moderate FAP expression, such as the uterus and breast, also showed higher FAPI tracer uptake in a recent study with SUVmax of 12.2 ± 7.3 and 4.5 ± 1.5, respectively, and this difference may be related to the biological age of women, and large sample studies are needed to confirm this finding (69, 73). Furthermore, FAPI-specific PET is still controversial in diagnosing bone metastases and lymph node metastases in different tumors (44, 74, 75), whether it is superior to ^18^F-FDG in diagnosing primary colorectal cancer and pancreatic cancer combined with chronic inflammation of the pancreas also needs further study (76). In another way, changes in the stroma during tumor development may lead to changes in the expression of FAP, so whether different aspects of tumor variability may have an impact on FAPI imaging results needs to be further explored. In brief, FAPIs cannot yet replace the work of FDG, but they can be used as a complement to it.

## Discussion

A total of 948 patients were included in the review, of which 891 patients were included in the malignancy imaging portion, including 598 Europeans, 287 Asians, and 1 North American, with an age range of 18–86 years. Existing studies did not find differences in the biodistribution of FAPI imaging agents between individuals, but age differences may have an impact on FAPI imaging results, e.g., the intensity of FAPI uptake in the uterus and breast is significantly higher in women of childbearing age than in women of non-childbearing age, which may pose a clinical challenge (57). The study is at a preliminary stage, and age, epidemiological factors and other factors that may contribute to differences in imaging results require further studies with large sample acquisition.

Although clinical studies of FAPI PET/CT are still at an early stage, mostly small sample size studies and lack of long-term follow-up data, and some clinical issues (e.g., tumor combined with inflammatory lesions and physiological uptake of organs that may be mixed with malignant lesions) need further clarification in subsequent studies. However, the light has just dawned, the available studies have shown that FAPIs are a promising class of tracers, and most of the FAPIs currently applied for imaging present relatively favorable pharmacokinetics and FAP affinity. In a variety of cancers such as glioma, gastrointestinal tumors, liver tumors, oral squamous carcinoma, and nasopharyngeal carcinoma, FAPIs are better than FDG for imaging, providing important molecular information and imaging basis for early diagnosis, precise staging, and guiding treatment. They also perform superiorly in non-malignant diseases (e.g., myocarditis and IgG_4_-RDs) and are expected to become the new molecule of the century.

Nineteen prospective studies designed to further explore the imaging characteristics of ^68^Ga-FAPIs are enrolling subjects in trials that will compare head-to-head with conventional imaging agents such as FDG, PSMA, FDOPA, and DOTATATE. These upcoming studies not only will include more types of tumors and expand the sample size of existing studies but will also perform immunohistochemical staining to correlate tracer uptake in tumors with FAP expression. While ^68^Ga-FAPI is the initial FAPI tracer used for PET imaging, both ^68^Ga and ^18^F-labeled FAPIs show more favorable imaging results, and the long half-life advantage demonstrated by ^18^F-labeled FAPIs is more favorable for its promotion in clinical applications.

We believe that FAPI imaging will play an important role in the identification of benign and malignant diseases, the accurate staging of malignant tumors, finding the primary foci of malignant tumors, monitoring the efficacy, identifying postoperative residual and fibrous tissue proliferation, assessing recurrence, finding biopsy sites where positive results can be more easily obtained, and guiding radiotherapy.

## Author Contributions

YH, LC, and RH conceived and designed the study. RH, YP, SH, CY, FY, YZP and JL retrieved and analyzed the documents. YP and SH collected the Figures. RH wrote the paper. YH and LC supervised the study, reviewed, and edited the manuscript. All authors contributed to the article and approved the submitted version.

## Funding

This study was Supported by the opening project of medical imaging key laboratory of Sichuan Province (MIKLSP202001), the National Natural Science Foundation of China (No. 81960496), Yunnan Fundamental Research Projects (No. 202101AT070050), National Natural Science Foundation of China (81901781), and Science and Technology Planning Project of Guangzhou, China (202102011130280027).

## Conflict of Interest

The authors declare that the research was conducted in the absence of any commercial or financial relationships that could be construed as a potential conflict of interest.

## Publisher’s Note

All claims expressed in this article are solely those of the authors and do not necessarily represent those of their affiliated organizations, or those of the publisher, the editors and the reviewers. Any product that may be evaluated in this article, or claim that may be made by its manufacturer, is not guaranteed or endorsed by the publisher.
